# Supplementary material for: From data to insights: a tool for comprehensive Quantification of Continuous Glucose Monitoring (QoCGM)
Source: PeerJ. 2025 Jun 9;13:e19501. doi: 10.7717/peerj.19501 (PMC12161138; doi:10.7717/peerj.19501)
Supplement: Supplemental Information 3 [file peerj-13-19501-s003.docx]

| Supplementary material S1 |
| --- |

Supplementary material S1 provide detailed explanation and mathematical definition of the CGM derived metrics.

| MAGE | Identify significant excursions $\lambda$ above a threshold $\sigma$.  $MAGE=\frac{1}{n}\sum_{i=1}^{n} \left\vert\lambda_{i} \right\vert if \left( \left\vert\lambda_{i} \right\vert\geq\sigma\right)$ |
| --- | --- |
| GRI | ${GRI}_{hypo}= VLow + (0.8 \cdot Low)$  ${GRI}_{hyper}= VHigh + (0.5 \cdot High)$  $VLow=\frac{1}{n}\sum_{i=1}^{n} \left\{ \begin{aligned} 1 \\ 0 \end{aligned} \right._{otherwise}^{if x_{i} <54 mg/dL}$  $Low=\frac{1}{n}\sum_{i=1}^{n} \left\{ \begin{aligned} 1 \\ 0 \end{aligned} \right._{otherwise}^{if x_{i} <70 \bigwedge x_{i}\geq54 mg/dL}$  $\mathrm{VHigh}=\frac{1}{n}\sum_{i=1}^{n} \left\{ \begin{aligned} 1 \\ 0 \end{aligned} \right._{otherwise}^{if x_{i}>250 mg/dL}$  $High=\frac{1}{n}\sum_{i=1}^{n} \left\{ \begin{aligned} 1 \\ 0 \end{aligned} \right._{otherwise}^{if x_{i} \leq250 \bigwedge x_{i}>180 mg/dL}$ |
| Time in range | $TIR=100\cdot\frac{1}{n}\sum_{i=1}^{n} \left\{ \begin{aligned} 1 \\ 0 \end{aligned} \right._{otherwise}^{if x_{i} \geq70\bigwedge x_{i}\leq180mg/dL}$ |
| Mobilty | $Mobility=\sqrt{\frac{\mathrm{variance}\left( \frac{\Delta x_{i}}{\Delta t} \right)}{\mathrm{variance}\left( x \right)}}$  $\Delta x_{i}=x_{i+1}-x_{i} for i=1,2,\ldots,n-1$ |
| CONGA | $CONGA=\sqrt{\frac{\sum_{i=1}^{n} \left( x_{i}-\bar{x} \right)^{2}}{n-1}}$ |
| LBGI  HBGI | Transform the glucose values:  $f\left( BG \right)=1.509\times\left( {\log\left( BG \right)}^{1.084} \right)-5.381$  Calculate the risk function:  $r\left( BG \right)=10\times\left( f\left( BG \right) \right)^{2}$  Determine the LBGI:  $LBGI=mean\left( r\left( BG \right)\times1\left\{ f\left( BG \right)<0 \right\} \right)$  Determine the HBGI:  $HBGI=mean\left( r\left( BG \right)\times1\left\{ f\left( BG \right)>0 \right\} \right)$ |
| GRADE | Calculate the GRADE score for each glucose value:  ${GRADE}_{i}=425\times{(log10(log10(\frac{{BG}_{i}}{18}))+0.16)}^{2}$  Overall GRADE score:  $GRADE=\frac{1}{n}\sum_{i=1}^{n} {GRADE}_{i}$  GRADE for Hypoglycemia:  $GRADEhypo=100\times\frac{\sum_{{BG}_{i<90}} {GRADE}_{i}}{GARDE}$ |
| DTpM | $DTpM=\frac{\sum_{i=1}^{n-1} \left\vert{BG}_{i-1}-{BG}_{i} \right\vert}{n\times fs}$ |
